# Supplementary material for: Early evolution of beetles regulated by the end-Permian deforestation
Source: eLife. 2021 Nov 8;10:e72692. doi: 10.7554/eLife.72692 (PMC8585485; doi:10.7554/eLife.72692)
Supplement: Supplementary file 1. [file elife-72692-supp1.docx]

**Supplementary File 1. Taxonomic revisions.**

Revisions for Taldycupedinae.

The subfamily Taldycupedinae Rohdendorf, 1961 was characterized by the absence of branching vein, 7 to 10 parallel windows punctures, main vein similar to the intermediate veins and some abdominal characters (*Rohdendorf et al., 1961*). Later, Ponomarenko revised the diagnosis of the family as ten window puncture rows, with additional cell rows basally in most cases and 3 window puncture rows behind A3 (*Ponomarenko, 1969*). And then Carpenter removed the character “3 window puncture rows behind A3” from the diagnosis (*Carpenter, 1992*). Moreover, some species reported by Ponomarenko are not consistent with the diagnosis of ten window puncture rows, such as *Tecticupes martynovi* and *Tychticupoides grjasevi* (*Ponomarenko, 1969*; *Aristov et al., 2013*). Thus, the number of window puncture rows is not a definite character for this family. The basal veins, especially those in the cubital area, are unique among stem groups (Tshekardocoleidae, Permocupedinae, and Taldycupedinae) as an important character which is absent in extant beetles. Thus, we consider that some taxa of Taldycupedinae without this character should be re-examined. Here, we re-examined Mesozoic Taldycupedinae and proposed that the following specimens should be removed from this subfamily.

1. *Mesothoris grandis* Dunstan, 1923

This species was reported from the Upper Triassic of Queensland, Australia (*Dunstan, 1923*). The base of the holotype is lacking. Thus, we cannot identify whether there are additional windows puncture rows basally. We consider this species cannot be definitely classified to Taldycupedinae.

2. *Mesothoris punctomarginum* Dunstan, 1923

This species was reported from the Upper Triassic of Queensland, Australia (*Dunstan, 1923*; *Kirejtshuk, 2020*). We found that the decorations of the elytron are not windows punctures but are similar to rounded tubercles (*Jell, 2004*). In addition, due to the lack of the base of the elytron, it is not clear whether there are additional cell rows basally, an important character for Taldycupedinae (*Ponomarenko, 1969*; *Carpenter, 1992*). Thus, we suggest that this species cannot be definitely classified in Taldycupedinae.

3. *Simmondsia cylindrica* Dunstan, 1923

This species was reported from the Upper Triassic of Queensland, Australia (*Dunstan, 1923*). We found that there are not distinct additional cell rows basally (*Jell, 2004*). Besides, there are less than three cell rows in the anal area (*Jell, 2004*). Thus, we consider this species cannot be definitely classified in Taldycupedinae.

4. *Simmondsia subpiriformis* Dunstan, 1923

This species was reported from the Upper Triassic of Queensland, Australia (*Dunstan, 1923*). We found that there are not distinct additional cell rows basally (*Jell, 2004*). In addition, the vein A3 is extended to the elytra apex, that is quite different from the short A3 in other Taldycupedinae (*Ponomarenko, 1969*; *Carpenter, 1992*). Thus, we consider this species cannot be definitely classified in Taldycupedinae.

5. *Wuchangia latilimbata* Hong, 1985

This species was reported from the Lower Jurassic of Hubei, China (*Hong, 1985a*). The base of the elytron is blurred, and it is not clear whether additional cell rows existed. Besides, this specimen lacks the short anal vein. Thus, we suggest that this species cannot be classified in Taldycupedinae.

6. *Yuxianocoleus hebeiense* Hong, 1985

This species was reported from the Lower Jurassic of Hubei, China (*Hong, 1985b*). Its cell punctures are small, similar to striae (small punctures in a thin furrow), not real windows punctures. Thus, we consider that this species cannot be classified as Taldycupedinae.

Revisions for Asiocoleidae.

The family Asiocoleidae contains 12 genera and 28 species from the Middle Permian to Late Triassic of Russia, China, Australia, Kazakhstan, Kyrgyzstan and Mongolia (*Ponomarenko, 2011*). Ponomarenko (2011) proposed that the family Tricoleidae should be considered a junior synonym of Asiocoleidae (*Ponomarenko, 2011*). However, we think it is incorrect to merge Tricoleidae with Asiocoleidae because there is no definite autapomorphy for combining these two families. Therefore, we retain these two families in our analysis. We attributed *Schizotaldycupes*, *Asiocoleus*, *Asiocoleopsis*, *Bicoleus*, and *Tetracoleus* in Asiocoleidae, a formal group that lacks detailed body characters.

Replacement name for *Uskatocoleus convexus* Ponomarenko, 2013.

We noticed that the species name *Uskatocoleus convexus* Ponomarenko, 2013 had already been used by Rohdendorf (*Rohdendorf et al., 1961*). Therefore, we proposed a new species name *Uskatocoleus ponomarenkoi* as a replacement name for the preoccupied and junior homonym *Uskatocoleus convexus* Ponomarenko, 2013. The new species name is named in honour of the palaeoentomologist A.G. Ponomarenko for his comprehensive and profound contribution to the taxonomy of fossil beetles.

**REFERENCES**

Rohdendorf BB, Becker-Migdisova EE, Martynova OM, Sharov AG. 1961. Paleozoic insects from the Kuznetsk Basin. Trudy Paleontologicheskogo Instituta AN SSSR 85: 1–701.

Carpenter FM. 1992. Treatise on Invertebrate Paleontology, Part R: Arthropoda 4: superclass Hexapoda 3/4 (Geological Society of America and the University of Kansas, Boulder, Colorado and Lawrence, Kansas, 655 pp).

Ponomarenko AG. 1969. Historical development of archostematan beetles. Trudy Paleontologicheskogo Instituta AN SSSR 125: 1–240.

Aristov DS, Bashkuev AS, Golubev VK, Gorochov AV, Karasev EV, Kopylov DS, Ponomarenko AG, Rasnitsyn AP, Rasnitsyn DA, Sinitshenkova ND, Sukatsheva ID, Vassilenko DV. 2013. Fossil insects of the Middle and Upper Permian of European Russia. Paleontological Journal 47: 641–832. DOI: https://doi.org/10.1134/S0031030113070010

Dunstan B. 1923. Mesozoic insects of Queensland Part I. Introduction and Coleoptera. Queensland Geological Survey Publication 273: 1–88.

Kirejtshuk AG. 2020. Taxonomic review of fossil coleopterous families (Insecta, Coleoptera). Suborder Archostemata: superfamilies Coleopseoidea and Cupedoidea. Geosciences 10: 73. DOI: https://doi.org/10.3390/geosciences10020073

Jell PA. 2004. The fossil insects of Australia. Memoirs of the Queensland Museum 50: 1–124.

Hong YC. 1985a. Early Jurassic fossil insects from Daye of Hubei Province. Professional Papers of Stratigraphy and Palaeontology 15: 181–187.

Hong YC. 1985b. New fossil insects of Xiahuayuan Formation in Yuxian County, Hebei Province. Bulletin of the Tianjin Institute of Geology and Mineral Resources 13: 131–138.

Ponomarenko AG. 2011. New beetles (Insecta, Coleoptera) from Vyazniki locality, terminal Permian of European Russia. Paleontological Journal 45: 414–422. DOI: https://doi.org/10.1134/S0031030111040095
