# Supplementary material for: Early evolution of beetles regulated by the end-Permian deforestation
Source: eLife. 2021 Nov 8;10:e72692. doi: 10.7554/eLife.72692 (PMC8585485; doi:10.7554/eLife.72692)
Supplement: Supplementary file 2. [file elife-72692-supp2.docx]

**Supplementary File 1. List of taxa used for the phylogenetic analysis.**

Order Megaloptera

Family Sialidae Leach, 1815

1. Genus *Sialis* Latreille, 1803

Family Chauliodidae Davis, 1903

1. Genus *Chauliodes* Latreille, 1796

Order Coleoptera

Suborder Polyphaga Emery, 1886

Family Hydrophilidae Latreille, 1802

1. Genus *Helophorus* Fabricius, 1775

Suborder Myxophaga Crowson, 1955

Family Torridincolidae Steffan, 1964

1. Genus *Torridincola* Steffan, 1964

Suborder Adephaga Clairville, 1806

Family Trachypachidae Thomson, 1857

1. Genus *Trachypachus* Motschulsky, 1845

Suborder Archostemata Kolbe, 1908

Family Cupedidae Laporte, 1836

1. Genus *Cupes* Fabricius, 1801

Family Ommatidae Sharp and Muir, 1912

1. Genus *Omma* Newman, 1839

Family Tshekardocoleidae Rohdendorf, 1944

1. Genus *Tshekardocoleus* Rohdendorf, 1944

Family Permocupedidae Martynov, 1932

Subfamily Permocupedinae Martynov, 1932

1. Genus *Permocupes* Martynov, 1932

Subfamily Taldycupedinae Rohdendorf, 1961

1. Genus *Taldycupes* Rohdendorf, 1961

Family Rhombocoleidae Rohdendorf, 1961

1. Genus *Rhombocoleites* Ponomarenko, 1969

Subfamily Triadocupedinae Ponomarenko, 1966

1. Genus *Triadocupes* Ponomarenko, 1966

Family Ademosynidae Ponomarenko, 1968

1. Genus *Chaocoleus* Ponomarenko, Yan & Huang, 2014

Family Phoroschizidae Bouchard and Bousquet, 2020

1. Genus *Dikerocoleus* Lin, 1982

Family Triaplidae Ponomarenko, 1977

1. Genus *Triaplus* Ponomarenko, 1977
