# Supplementary material for: Early evolution of beetles regulated by the end-Permian deforestation
Source: eLife. 2021 Nov 8;10:e72692. doi: 10.7554/eLife.72692 (PMC8585485; doi:10.7554/eLife.72692)
Supplement: Supplementary file 3. [file elife-72692-supp3.docx]

**Supplementary File 2. Characters used for the phylogenetic analysis.**

1. Externally visible membranes: (0) present; (1) absent.

2. Tubercles: (0) absent or very indistinct; (1) present.

3. Scale-like setae: (0) absent; (1) present.

4. Ocelli: (0) three; (1) absent.

5. Constricted neck and postocular extensions: (0) absent or indistinct; (1) present.

6. Supraantennal protuberance: (0) absent; (1) present as moderately distinct bulge; (2) present as strongly pronounced protuberance.

7. Supraocular protuberance: (0) absent; (1) present as moderately distinct bulge; (2) present as strongly pronounced protuberance.

8. Posteromesal protuberance: (0) absent; (1) present.

9. Posterolateral protuberance: (0) absent; (1) present.

10. Antennal groove on head; (0) absent; (1) below compound eye; (2) above compound eye.

11. Gular sutures: (0) complete, reaching hind margin of head capsule; (1) incomplete, not reaching hind margin of head capsule; (2) absent.

12. Shape of gula: (0) not converging posteriorly; (1) converging posteriorly.

13. Tentorial bridge: (0) present; (1) absent.

14. Posterior tentorial grooves: (0) externally visible; (1) not visible externally.

15. Anterior tentorial arms: (0) well developed; (1) strongly reduced or absent, not connected with posterior tentorium.

16. Frontoclypeal suture: (0) present; (1) absent.

17. Labrum: (0) free, connected with clypeus by membrane; (1) fused with head capsule.

18. Musculus labroepipharyngalis: (0) present; (1) absent.

19. Musculus frontolabralis: (0) present; (1) absent.

20. Musculus frontoepipharyngalis: (0) present; (1) absent.

21. Length of antenna: (0) not reaching mesothorax posteriorly; (1) very elongate, reaching middle region of body.

22. Number of antennomeres: (0) 13 or more; (1) 11 or less.

23. Location of antennal insertion on head capsule: (0) laterally; (1) dorsally.

24. Extrinsic antennal muscles: (0) four; (1) three; (2) two.

25. Shape of mandible: (0) short or moderately long, largely covered by labrum in repose (1) very elongate and protruding in resting position (3) vestigial.

26. Ventromesal margin of sculptured mandibular surface: (0) not reaching position of mandibular condyle; (1) reaching mandibular condyle.

27. Cutting edge of mandible: (0) horizontal (1) with three vertically arranged teeth.

28. Separate areas with different surfaces on ventral side of mandible; (0) absent; (1) present.

29. Deep pit in cranio-lateral area of ventral surface of mandible: (0) absent; (1) present.

30. Galea: (0) without globular distal galeomere and basal galeomere not slender and stalk-like; (1) stalk-like basal galeomere and globular distal galeomere; (2) absent.

31. Lacinia: (0) present; (1) absent.

32. Apical segment of maxillary palp: (0) with only one apical field of sensilla (campaniform sensilla) (1) with an apical and a dorsolateral field of sensilla.

33. Digitiform sensilla on apical maxillary palpomere: (0) absent; (1) present.

34. Pit containing sensilla on dorsolateral field of apical maxillary palpomere: (0) absent; (1) present.

35. Deep basal cavity of prementum: (0) absent; (1) present.

36. Lid-like ventral premental plate: (0) absent; (1) present.

37. Transverse ridge of prementum: (0) absent; (1) present.

38. Anterior appendages of prementum: (0) paired ligula; (1) ligula subdivided into many digitiform appendages; (2) absent.

39. Mentum: (0) distinctly developed; (1) vestigial or absent.

40. Musculus tentoriopharyngalis posterior: (0) moderately sized, not distinctly subdivided into individual bundles; (1) complex, composed of series of bundles, origin from the gular ridges or lateral gular region.

41. Propleural suture (0) present; (1) absent.

42. Exposure of propleura: (0) fully exposed, propleura reaches anterior margin of prothorax; (1) exposed, not reaching anterior margin of prothorax; (2) internalized.

43. Fusion of propleura and protrochantinus: (0) absent; (1) present.

44. Prosternal grooves for tarsomeres: (0) absent; (1) present.

45. Length of prosternal process: (0) not reaching beyond hind margin of procoxae, very short or absent; (1) reaching hind margin of procoxae.

46. Shape of prosternal process: (0) not broadened apically; (1) apically broadened and truncate.

47. Broad prothoracic postcoxal bridge: (0) absent; (1) present.

48. Mesocoxal cavities: (0) not bordered by metanepisterum; (1) bordered by metanepisternum.

49. Mesoventrite with anteromedian pit for reception of prosternal process: (0) absent or only very shallow concavity; (1) distinct, rounded groove; (2) large hexagonal groove.

50. Propleuro-mesepisternal locking mechanism: (0) absent; (1) propleural condyle and mesepisternal socket; (2) mesepisternal condyle and propleural socket.

51. Connection of meso- and metaventrite: (0) sclerites distinctly separated, connected by a membrane; (1) articulated but not firmly connected; (2) firmly connected between and within mesocoxal cavities.

52. Transverse suture of mesoventrite: (0) present; (1) absent.

53. Mesal coxal joints of mesoventrite: (0) present; (1) absent.

54. Shape of mesocoxae: (0) globular or conical; (1) with deep lateral excavation and triangular lateral extension.

55. Exposed metatrochantin: (0) present, distinctly developed; (1) indistinct or absent.

56. Shape of penultimate tarsomere: (0) not distinctly bilobed; (1) distinctly bilobed.

57. Forewings: (0) membranous; (1) transformed into sclerotized elytra.

58. Venation of forewings: (0) distinct, not arranged in parallel rows; (1) parallel arrangement of distinct longitudinal veins; (2) longitudinal veins very indistinct or absent.

59. Elytral sclerotization pattern: (0) with a pattern of unsclerotized window punctures; (1) entirely sclerotized.

60. Elytral apex: (0) distinctly reaching beyond abdominal apex posteriorly; (1) slightly reaching beyond abdominal apex posteriorly; (2) reaching abdominal apex or shorter.

61. Transverse folding mechanism of hind wings: (0) absent; (1) present.

62. Oblongum cell of hind wing: (0) closed cell not differentiated as oblongum cell; (1) oblongum present; (2) open or absent.

63. Abdominal sternite I: (0) exposed; (1) concealed under metacoxae, largely or completely reduced.

64. Median ridge on ventrite 1: (0) absent; (1) present.

65. Number of exposed abdominal sternites (excluding sternite I): (0) more than six; (1) six; (2) five.

66. Arrangement of abdominal sterna: (0) abutting, not overlapping; (1) tegular or overlapping.

67. Difference between main vein and interval vein: (0) significant; (1) unconspicuous.

68. More intensive rows of window punctures in the base of elytron (more than 2 rows): (0) present; (1) absent.

69. Braned vein: (0) present; (2) absent.

70. Head shape of later instars: (0) parallel-sided, slightly narrowing anteriorly, or evenly rounded; (1) transverse, strongly rounded laterally, greatest width near hind margin.

71. Posteromedian emargination of head capsule: (0) absent; (1) present.

72. Endocarina: (0) absent; (1) present, forked.

73. Frontal suture of second and third instars: (0) distinct; (1) indistinct or absent.

74. Stemmata: (0) more than one pair of stemmata; (1) one pair of stemmata or eyeless.

75. Length of antenna: (0) at least 20% of greatest width of head capsule; (1) less than 20% of greatest width of head capsule.

76. Antennal segments: (0) four or more; (1) three or less.

77. Shape of distal part of mandible: (0) less than three apices; (1) three apices.

78. Retinaculum: (0) present; (1) absent.

79. Shape of mola: (0) not quadrangular, not delimited by a distinct margin; (1) quadrangular and delimited by a distinct margin; (2) missing.

80. Ligula: (0) unsclerotized; (1) sclerotized, enlarged and wedge-shaped.

81. Mentum and submentum: (0) not fused; (1) fused and narrowed between maxillary grooves.

82. Prothorax: (0) as broad as following segments; (1) broader than following segments.

83. Leg segments: (0) six; (1) five.

84. Claws: (0) paired; (1) paired.

85. Abdominal segments I–III of later instars: (0) shorter than thorax; (1) longer than thorax.

86. Tergal ampullae: (0) absent; (1) present.

87. Ventral asperities: (0) absent; (1) present.

88. Lateral longitudinal bulge of abdominal segments I–VII: (0) absent; (1) present.

89. Sclerotized process of tergum IX: (0) absent; (1) present.

90. Eversible lobes of segment IX: (0) absent; (1) present.

91. Urogomphi: (0) absent; (1) present.

92. Segment X: (0) exposed; (1) not visible externally.

93. Larval habitat: (0) not associated with wood; (1) associated with wood.

contribution to the taxonomy of fossil beetles.
