# Supplementary material for: Early evolution of beetles regulated by the end-Permian deforestation
Source: eLife. 2021 Nov 8;10:e72692. doi: 10.7554/eLife.72692 (PMC8585485; doi:10.7554/eLife.72692)
Supplement: Supplementary file 4. [file elife-72692-supp4.docx]

**Supplementary File 3. Characters used for the morphospace analysis.**

1. Sides of elytron: (0) external margin more convex;(1) sutural margin more convex; (2) both similar.

2. Shape of window punctures: (0) polygonal; (1) round or oval; (2) without window punctures.

3. M vein and Cu vein: (0) forming X-shaped vein; (1) not forming X-shaped vein; (2) without veins.

4. Number of impression row(s): (0) without impression row; (1) 7 rows; (2) 8 rows; (3) 9 rows.

5. Striae of elytron: (0) striae with punctures; (1) striae without punctures; (2) elytron without striae.

6. Schiza present or absent: (0) present; (1) absent.

7. Number of puncture rows: (0) elytron without puncture row; (1) 3 rows; (2) 7 or 8 rows; (3) 12 rows.

8. Shape of elytron: (0) widest at base part of elytron; (1) middle of elytron widest; (2) the base and middle similarly wide; (3) widest at apex of elytron.

9. Short striae of the base of elytron: (0) present; (1) absent.

10. Fusion of striae in the termination of elytron: (0) fusion of striae present; (1) striae not fused at apex; (2) elytron without striae.

11. Striae elongated to the termination: (0) present; (1) absent; (2) elytron without striae.

12. Lateral location of schiza: (0) on the base of elytron; (1) on the middle of elyton; (2) on the apex of elytron; (3) without absent.

13. Denticles of elytral margin: (0) present; (1) absent.

14. Longitudinal location of schiza: (0) near the external margin; (1) on the middle of elytron; (2) near the sutural margin; (3) schiza absent.

15. Interval vein: (0) mainly zigzag vein; (1) mainly straight vein; (2) mixed with zigzag and straight veins; (3) without veins.

16. Difference between main vein and interval vein: (0): significant; (1) blurry; (2) without vein.

17. Elytral veins elongated to the termination (excluding C, SC, A vein): (0) present; (1) absent; (2) elytron without veins.

18. Elytron tail: (0) present; (1) absent.

19. Branches of vein: (0) present; (1) absent; (2) elytron without branches.

20. Number of tubercles rows: (0) tubercle rows absent; (1) less than or equal to 7 rows; (2) more than 7 rows.

21. Tubercles rows between ridges: (0) present; (1) tubercle rows present but without ridges; (2) elytron without tubercle row.

22. Elytra aspect ratio: (0) less than 3; (1) 3-4; (2) more than 4.

23. Tubercles: (0) present; (1) absent.

24. Discrete punctures (or pits): (0) present; (1) absent.

25. Epipleuron: (0) absent; (1) present.

26. Epipleural border: (0) absent; (1) present.

27. Sutural border: (0) present; (1) absent.

28. Number of puncture rows in epipleuron: (0) single row; (1) multiple rows; (2) epipleuron without puncture rows; (3) elytron without epipleuron.

29. Punctures (or pits) in epipleural border: (0) present; (1) absent; (2) elytron without epipleural border.

30. Punctures (or pits) in sutural border: (0) present; (1) absent; (2) elytron without epipleural border.

31. Number of striae: (0) less or equal to 10 rows; (1) more than 10 rows.

32. Ribs: (0) present; (1) absent.

33. Multiple rows of window punctures in base of elytron (more than 2 rows): (0) present; (1) absent; (2) elytron without window punctures.

34. Adjacent multiple short striae in base of elytron: (0) present; (1) absent; (2) elytron without striae.

35. Shallow striae: (0) present; (1) absent.
